# Blood DNA methylation biomarkers for cognitive decline in older adults with type 2 diabetes

**DOI:** 10.1002/alz.084359

**Published:** 2025-01-09

**Authors:** Sigalit Manzali, Ramit Ravona‐Springer, Jasmine Jacob‐Hirsch, Anthony Heymann, Michal S Beeri, Lior Greenbaum

**Affiliations:** ^1^ Department of Pathology, Sheba Medical Center, Ramat Gan Israel; ^2^ The Joseph Sagol Neuroscience Center, Sheba Medical Center, Ramat Gan Israel; ^3^ Sackler Faculty of Medicine, Tel Aviv University, Tel Aviv Israel; ^4^ Memory clinic and The Joseph Sagol Neuroscience Center, Sheba Medical Center, Israel, Ramat‐Gan Israel; ^5^ The Joseph Sagol Neuroscience Center, Sheba Medical Center, Tel Hashomer Israel; ^6^ Sheba Cancer Research Center (SCRC), Chaim Sheba Medical Center, Ramat Gan Israel; ^7^ Wohl Centre for Translational Medicine, Chaim Sheba Medical Center, Ramat Gan Israel; ^8^ Maccabi Health Services, Tel‐Aviv Israel; ^9^ Herbert and Jackeline Krieger Klein Alzheimer's Research Center, Rutgers Biomedical and Health Sciences, newark, NJ USA; ^10^ The Danek Gertner Institute of Human Genetics, Sheba Medical Center, Ramat Gan Israel

## Abstract

**Background:**

Type 2 diabetes (T2D) is a recognized risk factor for dementia. This study aimed to pinpoint blood DNA methylation biomarkers for cognitive decline in older adults with T2D by comparing those who developed dementia with those who remained cognitively normal during follow‐up

**Method:**

Illumina Infinium MethylationEPIC microarray was used for the initial 24 couples and Infinium HumanMethylationEPIC microarray version 2.0 for the subsequent 8 couples. Beta and p‐values were calculated using Partek genomic suite and SeSAMe for the respective microarray versions. Human CpG annotation manifests for both versions were utilized, and quantile normalization (Partek genomic suite) facilitated comparisons across different arrays. Functional analysis employed Ingenuity IPA.

**Result:**

The analysis covered 128 samples (32 converters and 32 matched non‐converters) at baseline and a 36‐month follow‐up. Methylation levels significantly differed between converters and non‐converters, with 950 sites altered in converters versus 25 in non‐converters (FDR corrected p value ≤0.05) during follow‐up. To identify potential biomarkers, methylation changes unique to baseline were examined. Of the 249,490 shared methylation sites, 526 were differentially methylated between converters and non‐converters at baseline (p‐value ≤0.05, fold change ≥1.5). Notably, 293 sites were altered solely at baseline, while 233 showed differential methylation at both baseline and 36‐months follow‐up. Among the 169 genes related to the 293 sites altered only at baseline, Ingenuity IPA analysis identified 29 genes linked to neurodegeneration and cognitive impairment (17%) and 38 genes associated with inflammation (22%). Another set of interest included 137 genes related to the 233 sites differentiating converters from non‐converters at both time points, with some genes implicated in neurotransmitter secretion and regulation of neuron differentiation.

**Conclusion:**

These initial findings indicate significant blood methylation changes early in cognitive decline among older adults with T2D. These alterations, if validated in additional cohorts, could serve as biomarkers for T2D‐related cognitive decline.

